# Machine learning prediction of metabolic-associated fatty liver disease in type 2 diabetes: Emphasizing data imputation and feature selection

**DOI:** 10.1371/journal.pone.0339580

**Published:** 2026-02-24

**Authors:** Zahra Khosravi, Farnaz Barzinpour, Soghra Rabizadeh, Manouchehr Nakhjavani, Alireza Esteghamati

**Affiliations:** 1 School of Industrial Engineering, Iran University of Science and Technology, Tehran, Iran; 2 Endocrinology and Metabolism Research Center (EMRC), Imam Khomeini Hospital Complex, Vali-Asr Hospital, Tehran University of Medical Sciences, Tehran, Iran; Universita degli Studi della Campania Luigi Vanvitelli Scuola di Medicina e Chirurgia, ITALY

## Abstract

Metabolic-Associated Fatty Liver Disease (MAFLD) is common among Type 2 Diabetes (T2DM) patients. The coexistence of these conditions increases the risk of MAFLD progression and diabetes complications. Detecting MAFLD early is challenging due to its asymptomatic initial stages. In this study, we aimed to develop a machine learning model to predict MAFLD in T2DM patients. We conducted a cross-sectional study on 3,654 Iranian T2DM patients using their demographic and lab data. This study involved thorough data preprocessing, including evaluating various imputation methods on simulated missingness in a complete subset of the dataset. Additionally, four feature selection methods were applied to eight machine learning models to identify the most effective predictive model. The XGBoost classifier without feature selection achieved the best performance, with an accuracy of 80.6% and an area under the receiver operating characteristic curve (AUC) of 88.9%.Notably, certain features, such as alanine aminotransferase (ALT), platelet count (PLT) and Vitamin D(VitD) influenced the predictive performance.

## Introduction

Metabolic-associated fatty liver disease (MAFLD), previously known as nonalcoholic fatty liver disease (NAFLD), is the most widespread liver disorder globally, impacting about 24% of people worldwide [1]. MAFLD encompasses a range of liver conditions, from fat accumulation to cirrhosis [2]. Previous research has revealed a higher prevalence of MAFLD among individuals with type 2 diabetes mellitus (T2DM) compared to the general population [3].The coexistence of these conditions increases the risk of progressing MAFLD stages and developing complications like cardiovascular and chronic kidney diseases [4].

In recent years, international experts redefined NAFLD as MAFLD, removing alcohol intake as a diagnostic criterion. The new criteria are centered on hepatic steatosis, which must be accompanied by one of the following: overweight/obesity, T2DM, or evidence of metabolic dysregulation [5]. Studies estimate that 50% to 70% of T2DM patients also have MAFLD [6]. In Iran, the prevalence of MAFLD among patients with type 2 diabetes has been reported at approximately65% (95% CI: 37–92%) which is consistent with global estimates [7]. Given the global rise in insulin-resistance–related disorders and the projected increase of T2DM to 578 million cases by 2030 [8], the burden of MAFLD is expected to grow accordingly.

Despite its clinical importance, MAFLD often remains undiagnosed in its early stages due to the lack of symptoms. This delay hinders timely intervention [9]. An accurate diagnosis can encourage the adoption of healthier lifestyle habits or the initiation of pharmacological treatments, which can prevent disease progression and, ultimately, cirrhosis [10].

For MAFLD diagnosis, liver biopsy offers the highest accuracy but is limited by cost and associated risks. Alternative methods, including imaging techniques, ultrasound examinations, and liver function tests, vary in accuracy. Ultrasound is often employed alongside liver function tests to enhance diagnostic precision [11]. Therefore, there is a growing need for cost-effective, accurate, and noninvasive approaches to identify MAFLD in high-risk groups such as patients with T2DM.

Machine learning (ML) techniques, which can uncover complex nonlinear relationships in clinical data, offer a promising framework for early MAFLD risk prediction. Previous research on fatty liver disease prediction has applied both image-based and structured data-driven ML approaches. Imaging studies using ultrasound, MRI, or biopsy data have achieved high accuracy with the aid of deep learning [12–15], but these methods remain costly, equipment-dependent, and impractical for routine T2DM screening. In contrast, structured data-based approaches that rely on demographic and biochemical variables offer broader clinical applicability. Several studies using such data have reported promising results, with ensemble models such as Gradient Boosting, XGBoost, and Random Forest generally outperforming traditional regression models [16–18]. However, most of these studies focused on NAFLD rather than the recently redefined MAFLD, and only a few included diabetic populations despite their higher disease risk.

Research specifically addressing MAFLD prediction in T2DM patients is still limited. Most existing work has concentrated on isolated biomarkers (e.g., ALT, vitamin D, ADRP, AIP) [19–22] or conventional regression methods, which often yield modest performance and lack generalizability. Moreover, preprocessing aspects such as missing-value imputation, feature selection, and hyperparameter tuning remain underexplored, even though they can strongly affect model accuracy and interpretability. These gaps highlight the need for studies that systematically optimize preprocessing pipelines and compare multiple ML algorithms to improve predictive performance for MAFLD in T2DM patients.

The present study aimed to develop and compare multiple machine learning models for predicting MAFLD among Iranian patients with type 2 diabetes, using readily available biochemical and demographic data from their routine clinical checkups. Unlike most previous MAFLD prediction studies in T2DM populations that focused on a single variable or employed only one machine learning algorithm, this work applied and compared several models, with particular emphasis on ensemble-based approaches. Furthermore, we focused on preprocessing strategies, including handling and simulating missing values to identify the most suitable imputation method, and systematically compared different feature selection techniques to achieve a robust and clinically meaningful predictive outcome for MAFLD in patients with T2DM.

## Methodology

**Fig 1:**
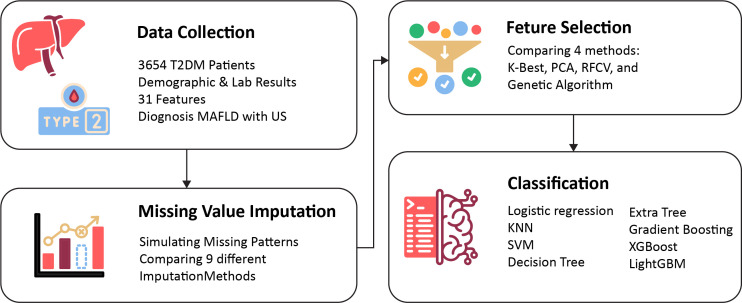
Framework of proposed methodology.

This study presents a ML framework for predicting MAFLD in T2DM patients. Fig 1 illustrates the structure of the proposed methodology.

### Data description

Data was collected over 10 years (2011–2021) from patients at Imam Khomeini Hospital in Tehran, Iran. The dataset initially comprised 67 features and 6403 records, including prescription details and dates related to various conditions. The full list of variables and their corresponding details is provided in Supplementary S1 Table. All patients in this dataset were diagnosed with T2DM, based on criteria from the American Diabetes Association (ADA) [23]. Diagnostic tests included fasting blood sugar (FBS), HbA1c, the 2-hour postprandial glucose test (2HPP), and the glucose tolerance test (GTT). Patients were classified as having T2DM if they exhibited HbA1c levels ≥ 6.5%, FBS levels ≥ 126 mg/dl, or 2HPP levels ≥ 200 mg/dl.

Participants were non-pregnant, without serious liver-related diseases, and over 20 years old. To ensure accurate fatty liver diagnosis, only patients who had undergone abdominal ultrasonography were included, reducing the dataset to 3964 records.

This study was approved by the Research Ethics Committee of Tehran University of Medical Sciences (Approval ID: IR.TUMS.IKHC.REC.1400.221). Written informed consent was obtained from all participants prior to their inclusion in the study. All data were anonymized prior to analysis, and the study adhered to the ethical standards for research involving human subjects. The dataset was accessed for research purposes on 18 Feburary 2023. All data were fully anonymized prior to access, and none of the authors had access to personally identifiable information at any stage during or after data collection.

### Data cleaning

Initially, irrelevant features were removed based on expert opinion to focus on relevant clinical variables. Acceptable ranges for each variable were determined, and values outside these ranges were treated as missing. Features with more than 45% missing values and rows with more than 50% missing values or duplicates were excluded. This resulted in 3769 records and 31 variables, plus the target variable (MAFLD). The robustness of these thresholds was further assessed through a sensitivity analysis using alternative cutoffs (30% and 60%), which demonstrated no material differences in cohort characteristics across scenarios (all standardized mean difference (SMD) < 0.01, Kolmogorov–Smirnov (KS) p = 1.00; see Supplementary S2 Table).

The features included demographic characteristics: sex, age, height, weight, waist circumference, hip circumference (Hip), and body mass index (BMI); medical history: diabetic duration (DDM), retinopathy (Retino), hypertension (HTN), cerebrovascular accident (CVA), and coronary artery disease (CAD). CAD was defined as having percutaneous coronary intervention (PCI), coronary artery bypass graft (CABG), or angioplasty. Additionally, features based on medical test results included: platelet count (PLT), diastolic blood pressure (DBP), C-reactive protein (CRP), vitamin D (VitD), insulin, total cholesterol (Chl), HDL cholesterol, LDL cholesterol, triglycerides (TG), aspartate aminotransferase (AST), ALT, alkaline phosphatase (ALKP), homeostatic model assessment for insulin resistance (HOMA), smoking status, fasting blood sugar (FBS), 2-hour postprandial blood sugar (2HPP), HbA1c, creatinine (Cr), and uric acid (UA).

### Machine learning methods

Eight machine learning algorithms were implemented and compared for MAFLD prediction: Logistic Regression (LR), Support Vector Machine (SVM), Decision Tree (DT), Random Forest (RF), Gradient Boosting (GB), XGBoost, LightGBM, and K-Nearest Neighbors (KNN). These models were selected to represent a range of linear, tree-based, and ensemble approaches commonly used in biomedical prediction tasks.

Model performance was assessed using accuracy, precision, recall (sensitivity), F1-score, and the area under the receiver operating characteristic curve (AUC). In addition, the R² (coefficient of determination) was used to evaluate the goodness of fit for imputation models during the preprocessing stage. For each classifier, performance metrics were computed as the mean and 95% confidence interval (CI) across the five cross-validation folds.

Descriptions of the models and the mathematical definitions and formulae for all evaluation metrics (Accuracy, Precision, Recall, F1, AUC, and R²) are provided in Supplementary S3 and S4 Tables.

### Handling missing values

**Fig 2:**
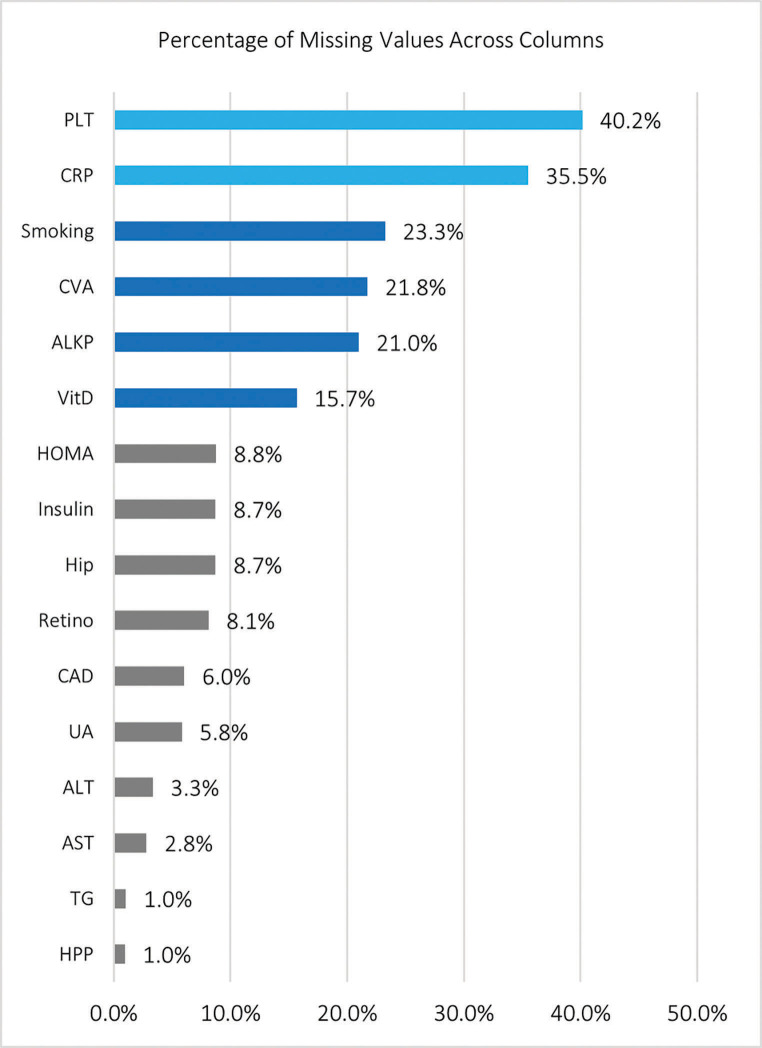
Percentage of missing values for each variable with missing data.

After cleaning the dataset, 25 out of 31 variables had missing values which are shown in Fig 2. Features without missing values included sex, age, height, weight, diastolic blood pressure, and BMI.

Various approaches exist for imputing missing data. Some methods are univariate, meaning they rely solely on the values within each feature independently to estimate missing values. Examples include the Mean and Median imputation techniques, where missing values in a feature are replaced by the mean or median of the observed values in that same feature. In contrast, multivariate methods leverage the values of other variables to predict missing data [24]. Selecting an appropriate imputation method requires an understanding of the nature of missing data, as the relationship between missingness and observed values influences the choice of approach. Based on the literature, missing data can be categorized into three types [25]:

Missing Completely at Random (MCAR): In this category the missingness is entirely random, and every record has an equal chance of having missing data in any variable.

Missing at Random (MAR): The probability of a missing value in a variable depends on other observed variables but is independent of the actual missing value.

Missing Not at Random (MNAR): The missingness is related to the unobserved value itself. For example, older individuals may be less likely to report their age.

In this study, data collection was conducted systematically by medical staff, meaning that the values of the records themselves were unlikely to influence the missingness mechanism. Consequently, the probability of MNAR is low. Instead, there remains a possibility that the missingness follows the MAR pattern, which can be statistically tested.

To determine the missingness mechanism in the dataset, Little’s MCAR test was applied. This statistical test first identifies distinct missingness patterns, i.e., which features had missing values simultaneously and then assesses whether the patterns occur randomly using a chi-square test [26]. The results (chi-square value = 6,987, degrees of freedom = 3,832, p-value ≈ 0) rejected the MCAR hypothesis, indicating that the data are MAR. This finding suggests a relationship between the probability of missingness and the values of other observed variables. Since the missing data mechanism was assumed to be MAR, imputations were conditioned on other observed variables. Therefore, the marginal distributions of variables may differ after imputation, which is expected and does not indicate bias under the MAR assumption.

Based on this result, univariate methods may not be suitable, as they do not account for interdependencies between variables. Instead, multivariate imputation methods, which consider relationships among multiple variables, were considered more appropriate for handling missing values in this study.

### Types of missing values

Given that the missing data were MAR, multivariate imputation methods were employed to estimate the missing values more accurately. Three popular techniques were utilized:

1. Multiple Imputation by Chained Equations (MICE): MICE is an iterative method for handling missing data by generating multiple imputations for each missing value. It uses a series of predictive models to estimate missing values based on other observed variables in the dataset. A key advantage of MICE is its flexibility in model selection, allowing users to specify different machine learning algorithms for each variable during the imputation process, making it adaptable to various data characteristics [24].
2. K-Nearest Neighbors (KNN) Imputer: The KNN imputer estimates missing values by identifying the ‘k’ instances in the dataset that are most like the instance with missing data, based on a defined distance metric (e.g., Euclidean distance). For a given missing value, the algorithm imputes it using the mean (for continuous data) or mode (for categorical data) of these ‘k’ nearest neighbors. This method leverages the similarity between instances to provide accurate imputations [27].
3. Missforest: Missforest is a non-parametric imputation method that utilizes random forests to predict missing values. It can handle both continuous and categorical data and is capable of capturing complex interactions between variables without requiring explicit model specification. The algorithm iteratively trains a random forest model on the observed data to predict missing values, updating the imputed dataset until convergence is achieved [28].

### Missing value simulation

Since the actual missing values in the dataset are unknown, an effective approach for evaluating different imputation methods is to simulate missingness patterns [25]. The results of Little’s MCAR test indicate that the MAR hypothesis is rejected, meaning there is a relationship between the probability of missingness and the values of other features. Therefore, a simulation process was designed to replicate this pattern.

To conduct this process, a subset of the dataset where all values were available was first selected, ensuring that no missing values existed initially. Within this subset, null values were artificially introduced, and various imputation methods were then applied to fill in the missing data. Since the original values were known, the imputed results could be compared against the actual values, allowing for the determination of the most effective imputation method.

To ensure that the simulated missing values closely resembled real missingness patterns, observed relationships between missing and non-missing values in the full dataset were used. The process involved selecting one column at a time as the target column, where missing values were labeled as “1” and observed values as “0.” A classifier was then trained using the remaining columns as input features to predict the probability of missingness in the target column. This process was repeated for all columns containing missing values. Multiple classifiers were trained for each column, and the best-performing model was chosen based on its ability to predict missing data. As a result, each column with missing values was assigned an optimal classifier that best modeled its missingness pattern. For instance, in the dataset with 31 columns, 25 columns contained missing values. Each of these 25 columns was iteratively selected as the target column, where missing and observed values were encoded as “1” and “0,” respectively. The remaining 30 columns were used as input features for training classifiers to predict missingness probability. By the end of this step, 25 trained classifiers had been obtained, one for each column with missing values.

Next, these trained classifiers were applied to the complete dataset (which initially had no missing values) to simulate missingness. Again, each column was chosen as the target, and its corresponding trained classifier was used to predict the probability of missingness for each record based on the values of other columns. To ensure that the missing values reflected real-world distributions, the missingness ratio observed in the full dataset was replicated. For example, if a column originally had 20% missing values, then in the simulated subset, the top 20% of records with the highest predicted missingness probability were artificially set to null.

By repeating this process for all columns, a simulated missing dataset was generated that closely mirrored the real missingness patterns, providing a robust foundation for evaluating different imputation methods.

**Fig 3:**
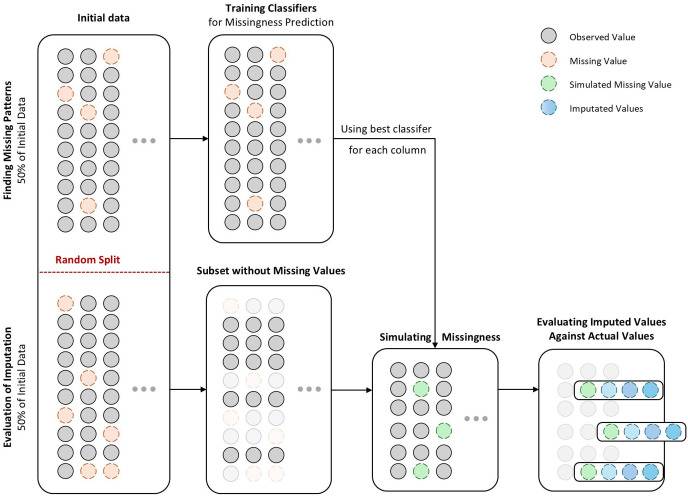
The process of simulating missingness in the dataset.

The process has been presented in Fig 3. To implement the described method on our dataset, the data was divided into two halves. The first half was used to extract a fully observed subset by removing all rows containing missing values; this ensured a complete dataset for simulating missingness. The second half was used to develop models trained to predict the probability of missingness based on observed values in other columns. To ensure the rigor and stability of the results, the entire simulation procedure, from data splitting through imputation evaluation, was repeated five times using different random seeds.

### Prediction of probability of missingness

Eight classifiers were chosen for this task, including four basic models and four ensemble models: LG, KNN, SVM, DT, RF, ET, AdaBoost, and XGBoost.

Hyperparameter tuning was conducted using Grid Search with cross-validation to optimize each classifier’s performance. Grid Search cross-validation (CV) is a systematic approach to hyperparameter tuning in which a predefined grid of hyperparameter values is exhaustively searched [29]. Each classifier was trained and tuned for columns with missing values, and the best classifier was selected based on the F1. The F1-score was chosen due to the imbalance in missingness across columns, ensuring unbiased classification.

### Evaluating imputation methods

To simulate missingness in the second half of the dataset, the best classifier was used for each variable to predict the probability of missing data. Nine imputation methods were evaluated, including different implementations of MICE with estimators such as Ridge Regression, SVM, DT, RF, ET, AdaBoost, and XGBoost, along with KNN and missforest. The imputation methods were assessed by calculating R² for continuous variables and Accuracy for binary variables. A weighted average was computed for each metric, considering the proportion of missing values in each variable. This provided an overall performance measure for each imputation method, aiding in identifying the most effective one. The complete hyperparameter grids and the best-performing configurations of all imputation models, are provided in Supplementary S5 Table.

### Outlier detection

After data imputation, outlier detection was performed to eliminate noise from incorrectly imported data while retaining valuable outlier values. Outliers were identified after imputation, as this ensured that true missingness patterns did not affect the detection process.

Despite the typical use of statistical approaches in medical studies [30], given the unknown distribution of the dataset, a density-based method, Local Outlier Factor (LOF), was used. LOF detects outliers by assessing the local density of each data point relative to the densities of its neighboring points. It computes a score that reflects how isolated a point is relative to its surrounding points; a significantly lower local density suggests that the point is an outlier [31]. This method detected 29 records as outliers, which were removed due to apparent data issues.

### Balancing the dataset

After outlier removal, 3740 records remained, 1827 (48.8%) in class 1 (MAFLD) and 1913 (51.1%) in class 0 (non-MAFLD), indicating a slight imbalance. Using an unbalanced dataset can bias the machine learning models toward the majority class. Therefore, the dataset was balanced by randomly removing 86 rows from class 0, resulting in a final dataset of 3654 records.

### Training and testing procedure

The dataset was first divided into independent training (80%) and test (20%) subsets. All model development steps were performed exclusively on the training data. Within the training subset, a stratified 5-fold cross-validation was used to conduct hyperparameter tuning through grid search. After obtaining the optimal hyperparameters, feature selection was carried out on the entire training set. The trained models were then re-evaluated within the training data using 5-fold cross-validation to ensure robust estimation. The independent test set was kept completely unseen during these steps and was used only for the final evaluation and comparison of models.

### Feature selection

To identify the most relevant variables for predicting MAFLD, a diverse set of feature selection methods was intentionally chosen to encompass different approaches, including filter-based (SelectKBest), wrapper-based (Recursive Feature Elimination with Cross-Validation), feature extraction (Principal Component Analysis), and a metaheuristic-based approach (Genetic Algorithm). This selection was motivated by the goal of exploring and comparing distinct methodologies, ranging from statistical filtering to iterative optimization, to ensure a comprehensive evaluation of feature importance and enhance the robustness of the predictive model.

Select K Best: Select K Best is a univariate feature selection technique that ranks features based on their individual relationship with the target variable using a specified scoring function. Common scoring metrics include the ANOVA F-test for numerical input features and mutual information for categorical variables. The top K features with the highest scores are retained for further analysis. This method is computationally efficient and widely used in preliminary feature selection steps [32].

Principal Component Analysis (PCA): PCA is an unsupervised technique that reduces the dimensionality of the input data by projecting the original features onto a new set of uncorrelated axes, referred to as principal components. These components are ranked by the amount of variance they capture in the dataset, with the first few components preserving most of the information. PCA is particularly useful for addressing multicollinearity, reducing noise, and improving model efficiency, though it may lead to a loss of interpretability since transformed features are linear combinations of the original ones [33].

Recursive Feature Elimination with Cross-Validation (RFECV): RFECV is a wrapper-based feature selection method that iteratively eliminates the least important features, optimizing model performance through cross-validation. In each iteration, a machine learning algorithm is trained, and based on metrics like coefficient weights the feature with the lowest importance is eliminated. These iterations continue until the best possible subset of features is selected. RFECV incorporates cross-validation to ensure robustness, making it a reliable method for selecting highly predictive feature [34].

Genetic Algorithm (GA) for Feature Selection: GA is a metaheuristic optimization technique inspired by natural selection. It encodes potential solutions (feature subsets) as chromosomes, which undergo evolutionary processes such as crossover (combining parts of different solutions) and mutation (introducing small random changes). Over successive generations, individuals with higher fitness, determined by an evaluation function measuring model performance, are more likely to be selected. This iterative process leads to the discovery of an optimal or near-optimal feature subset [35].

K-fold cross-validation was used for training. For K Best and PCA, tuning involved varying the number of output features. The Genetic Algorithm’s hyperparameters were optimized using the Taguchi method, a design-of-experiments approach that systematically evaluates parameter combinations to identify robust settings with minimal computational cost [36]. Python 3, along with numpy, pandas, scikit-learn, SciPy, XGBoost, and lightgbm, was employed for data processing and classification.

### Classification

After completing the preprocessing steps, several machine learning algorithms were applied to classify patients with and without MAFLD. The baseline models included LG, KNN, SVM, and DT. To enhance performance, ensemble methods, which combine multiple models, were employed. The DT model was selected as the base model due to its interpretability and frequent use in ensemble methods. While Decision Trees are prone to overfitting, performance evaluation using cross-validation indicated that overfitting was not a major concern in this case, potentially due to appropriate hyperparameter tuning. Consequently, the focus was placed on boosting methods rather than bagging, as boosting can enhance predictive accuracy by sequentially improving weak learners, whereas bagging primarily alleviates overfitting by reducing variance. [37]. Therefore, ET was chosen as a bagging method, while GB, XGBoost, and LightGBM were selected as boosting methods.

The complete hyperparameter grids and the best-performing configurations of all classification models, are provided in Supplementary S6 Table.

To ensure the robustness and reproducibility of the classification results, the entire model training and evaluation process was repeated ten times under different random seeds. The reported results represent the mean and 95% confidence intervals computed across these ten independent runs.

## Results

This section presents the findings of this study, organized into distinct subsections.

### Variables

After data cleaning, 31 features remained, as shown in Table 1 (continuous variables) and Table 2 (binary variables). The Shapiro-Wilk test indicated that none of the continuous variables followed a normal distribution; Consequently, the Kruskal-Wallis test was used to assess the significance of the relationship between MAFLD and each continuous variable. For binary variables, the chi-square test was employed to evaluate their significance.

**Table 1:** Details and characteristics of continuous variables for patients with MAFLD and patients without MAFLD.

Characteristic	non-MAFLD Value (Mean, Q1 - Q3)	MAFLD Value (Mean, Q1 - Q3)	P-value
Age, years	60.4 (53-68)	55.3 (47-64)	<0.001
ALKP, U/L	146.6 (100-173)	162.9 (112-200)	<0.001
ALT, U/L	21.5 (15-25)	38 (24.5-48)	<0.001
AST, U/L	19.2 (15-22)	28 (19-33)	<0.001
BMI	28.5 (25.1-31.2)	31.1 (27.2-34.3)	<0.001
Chl, mg/dL	171.4 (140-198.5)	181.1 (148-208)	<0.001
Cr, mg/dL	1 (0.8-1.1)	1 (0.9-1.1)	0.6048
CRP, mg/L	1.4 (1-1.4)	1.8 (1-1.9)	<0.001
DBP, mmHg	76.8 (70-80)	79 (75-80)	<0.001
DDM, years	11.4 (4-17)	10.3 (4-15)	<0.001
FBS, mg/dL	157.5 (121.5-182)	158.1 (121-179.5)	0.7399
HBA1C	7.6 (6.5-8.5)	7.6 (6.5-8.5)	0.5862
HDL, mg/dL	44.9 (37-52)	43.9 (37-50)	0.0019
Height, cm	163.3 (156-170)	165.2 (158-173)	<0.001
Hip, cm	104.7 (100-109)	109 (102-114)	<0.001
HOMA	3.8 (2.5-4.5)	4.6 (2.8-5.5)	<0.001
2HPP, mg/dL	210.9 (154.5-250)	213.3 (150-255)	0.6392
Insulin, µIU/mL	9.7 (8-10.3)	12.1 (8.1-14)	<0.001
LDL, mg/dL	95.6 (71-117)	102 (77-124)	<0.001
PLT, cells/µL	247.5 (226.1-267.8)	266.9 (249.3-295)	<0.001
Tg, mg/dL	160.7 (103.5-195)	182.6 (115-226)	<0.001
UA, mg/dL	5 (4-5.6)	5.3 (4.2-6)	<0.001
VitD, ng/mL	26.9 (20-30)	23.5 (16.1-28.1)	<0.001
Waist, cm	98.1 (91-103)	103.2 (96-110)	<0.001
Weight, Kg	75.8 (67-83)	84.8 (73-94)	<0.001

ALKP, Alkaline Phosphatase; ALT, Alanine Aminotransferase; AST, Aspartate Aminotransferase; BMI, Body Mass Index; Chl, Cholesterol; Cr, Creatinine; CRP, C-Reactive Protein; DBP, Diastolic Blood Pressure; DDM, Duration of Diabetes Mellitus; FBS, Fasting Blood Sugar; HBA1C, Hemoglobin A1c; HDL, High-Density Lipoprotein; Hip, Hip Circumference; HOMA, Homeostasis Model Assessment; 2HPP, 2-Hour Postprandial Glucose; LDL, Low-Density Lipoprotein; PLT, Platelet Count; Tg, Triglycerides; UA, Uric Acid; VitD, Vitamin D; Waist, Waist Circumference

**Table 2:** Details and characteristics of binary variables for patients with MAFLD and patients without MAFLD.

Characteristic	non-MAFLD % (n)	MAFLD % (n)	P-value
CAD	12.2% (460)	9.9% (372)	0.0095
CVA	1.1% (43)	0.6% (22)	0.0165
HTN	21.3% (803)	18.9% (712)	0.0794
Retino	6.0% (227)	4.4% (164)	0.0021
Sex			<0.001
Female	49% (903)	58.4% (1125)	
Male	51% (941)	41.5% (800)	
Smoking	1.9% (73)	1.6% (60)	0.4732

CAD, Coronary Artery Disease; CVA, Cerebrovascular Accident (Stroke); HTN, Hypertension; Retino, Retinopathy

### Evaluation of imputation methods

**Fig 4:**
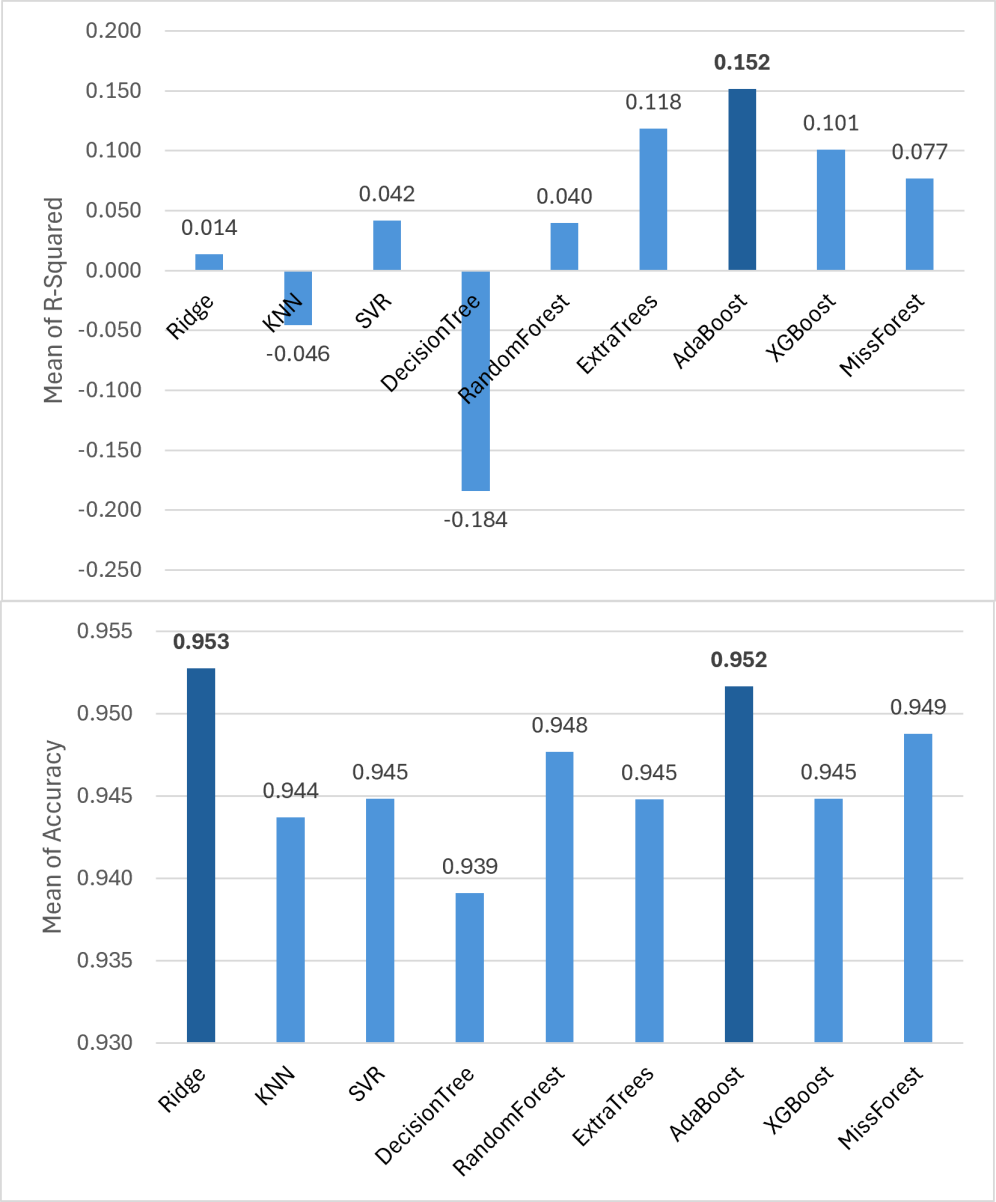
Performance comparison of different imputation methods for binary and continues variables (mean results over five runs).

After simulating the missingness in a complete subset of the dataset, the performance of imputation methods was evaluated. This process was repeated five times, and the mean results across these runs are presented in Fig 4.

For continuous features, AdaBoost achieved the highest performance. For binary features, all imputers yielded comparable results, with Ridge having the highest accuracy along with Adaboost (with 0.001 difference). Therefore, AdaBoost was selected as the final imputation method, and all subsequent analyses were performed on the dataset imputed using this approach.

### Feature selection and classification

The accuracy of different feature selection strategies compared with baseline models using all features is summarized in Table 3. Results are reported as mean accuracy ± standard deviation (SD) and 95% confidence intervals (CI) across five cross-validation folds. The bolded values indicate the highest mean accuracy for each classifier.

**Table 3:** Accuracy and number of selected features for each classifier with various feature selection methods.

Classifiers	Without Selection	K Best		PCA		RFCV		GA	
		n	Accuracy	n	Accuracy	n	Accuracy	n	Accuracy
Logistic regression	0.765 ± 0.014 [0.761,0.769]	26	0.765 ± 0.015 [0.761,0.769]	30	0.765 ± 0.014 [0.761,0.769]	30	0.764 ± 0.014 [0.758,0.769]	17	**0.766 ± 0.014 [0.763,0.77]**
KNN	0.724 ± 0.016 [0.72,0.728]	12	0.751 ± 0.016 [0.747,0.756]	15	0.733 ± 0.015 [0.729,0.737]	30	0.723 ± 0.019 [0.716,0.731]	9	**0.763 ± 0.015 [0.759,0.767]**
SVM	0.768 ± 0.012 [0.765,0.771]	**18**	**0.768 ± 0.013 [0.765,0.772]**	30	0.768 ± 0.012 [0.765,0.772]	30	0.765 ± 0.014 [0.759,0.77]	31	0.74 ± 0.014 [0.736,0.744]
Decision Tree	0.747 ± 0.017 [0.743,0.752]	8	0.745 ± 0.016 [0.74,0.75]	14	0.695 ± 0.019 [0.69,0.7]	30	0.745 ± 0.02 [0.737,0.753]	15	**0.746 ± 0.016 [0.741,0.75]**
Extra Tree	0.79 ± 0.014 [0.786,0.794]	24	0.791 ± 0.015 [0.787,0.795]	28	0.77 ± 0.013 [0.767,0.774]	30	0.788 ± 0.016 [0.782,0.794]	11	**0.794 ± 0.013 [0.79,0.798]**
Gradient Boosting	0.803 ± 0.015 [0.799,0.807]	22	**0.803 ± 0.014 [0.799,0.807]**	30	0.763 ± 0.017 [0.758,0.768]	30	0.801 ± 0.015 [0.795,0.807]	14	0.799 ± 0.014 [0.795,0.803]
XGBoost	**0.806 ± 0.014 [0.802,0.81]**	31	0.802 ± 0.015 [0.798,0.806]	25	0.759 ± 0.015 [0.755,0.763]	30	0.801 ± 0.014 [0.796,0.807]	24	0.804 ± 0.017 [0.799,0.809]
LightGBM	0.803 ± 0.014 [0.799,0.806]	30	0.799 ± 0.014 [0.795,0.803]	30	0.765 ± 0.014 [0.761,0.769]	30	0.801 ± 0.014 [0.796,0.807]	18	**0.803 ± 0.015 [0.799,0.807]**

PCA, Principal Component Analysis; RFECV, Recursive Feature Elimination with Cross-Validation; GA, Genetic Algorithm; KNN, K-Nearest Neighbors; SVM, Support Vector Machine; XGBoost, eXtreme Gradient Boosting; LightGBM, Light Gradient Boosting Machine

Among them, XGBoost without Overall, the ensemble models demonstrated very similar performance, with only marginal differences across algorithms and feature selection approaches. feature selection achieved the highest overall accuracy (0.806 ± 0.014) and was therefore identified as the best-performing model. The Gradient Boosting and LightGBM models followed closely, showing only slight differences in performance. For these two models, the versions incorporating feature selection methods, K Best for Gradient Boosting and Genetic Algorithm for LightGBM, yielded nearly identical results to their corresponding models without feature selection. Given these minimal gaps, the feature-selected versions were considered representative of their best performance configurations

**Fig 5:**
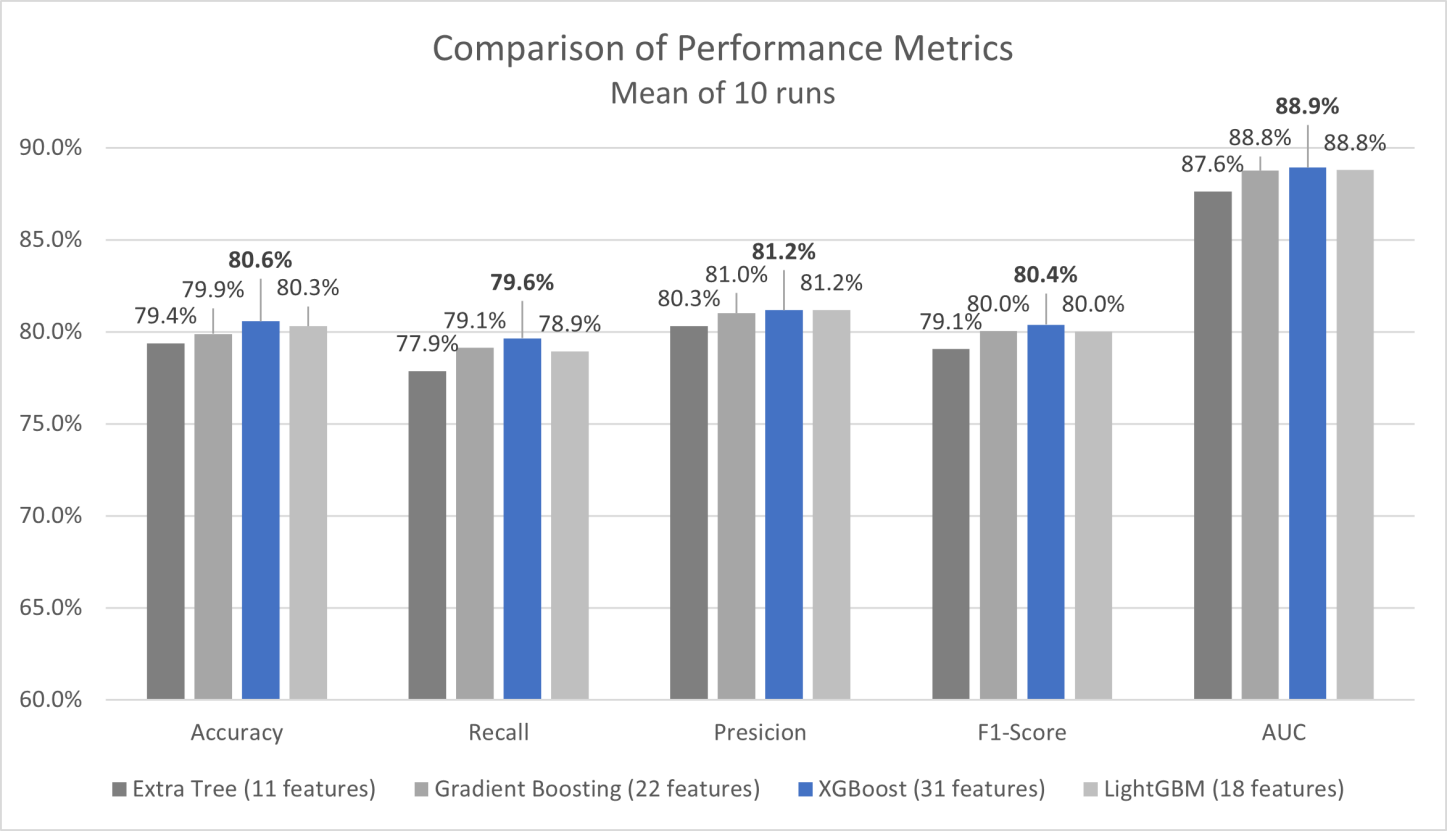
Comparison of performance metrics for ensemble methods.

Using the preferred feature selection method for each classifier, the mean of outcomes for the ensemble methods, which outperformed the basic models, are illustrated in Fig 5. The figure presents a comparison of various metrics, including Accuracy, Recall, Precision, F1-score, and the AUC.

### Feature importance

**Fig 6:**
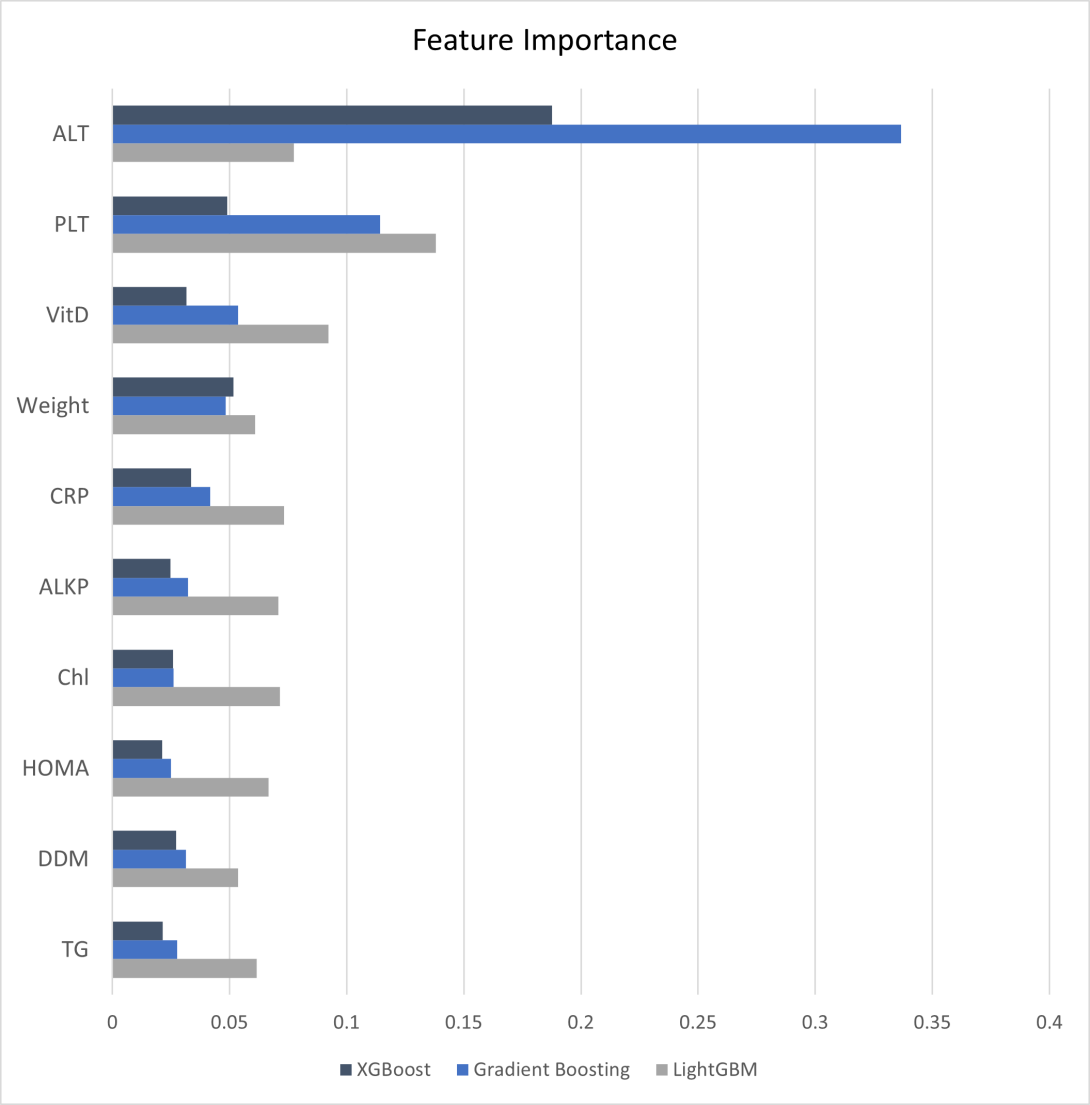
Top ten features with the highest mean importance across XGBoost, LightGBM, and Gradient Boosting models.

Fig 6 illustrates the top ten features with the highest mean importance scores across the three best-performing models, XGBoost, LightGBM, and Gradient Boosting. The bar plot shows, for each of these top features, their relative importance within each model. The full values are presented in Supplementary S7 Table.

**Fig 7:**
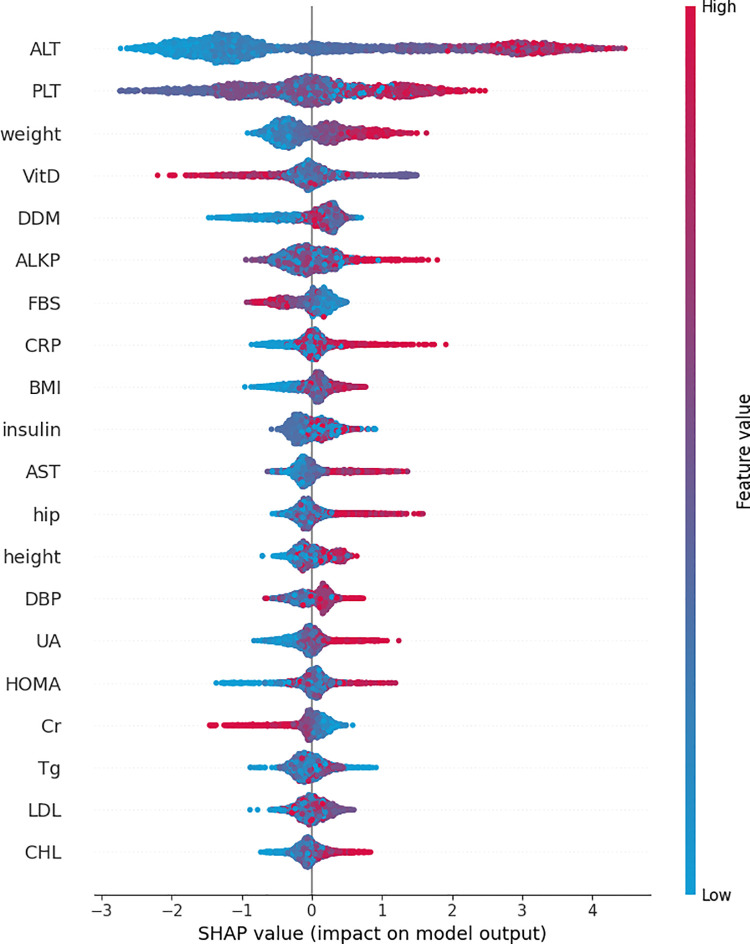
SHapley Additive exPlanations value plot of different features in XGBoost classifier.

To further analyze the data, a SHapley Additive exPlanations (SHAP) plot using the XG Boost model is presented in Fig 7. SHAP plots, derived from SHAP values, illustrate the contribution of each feature to the prediction (e.g., class membership probability) and how these contributions deviate from the mean prediction across all data points [38].

## Discussion

This study utilized data from 3,769 Iranian T2DM patients to develop machine learning models for predicting the presence of MAFLD. Different feature selection methods were applied during preprocessing. Given the relatively small number of variables (n = 31) and the fact that ensemble algorithms such as Gradient Boosting, XGBoost, and LightGBM inherently perform internal feature weighting, the added predictive value of explicit feature selection was limited. Nevertheless, these methods were included because of their practical and interpretative benefits, including: (1) reducing computational costs, (2) simplifying model deployment for medical staff by minimizing required input variables, and (3) improving understanding of variable relevance and model robustness [39]. Although feature selection decreased the number of predictors, its impact on the model’s performance was minimal, indicating the XGBoost model can effectively handle redundant variables and maintain stable accuracy without explicit feature reduction.

After comparing different classifiers and different feature selection methods, the most important features in predicting MAFLD in patients with T2DM were identified checking the importance of the features in the three models with higher accuracy. Based on that the most important features were ALT, PLT, VitD, Weight and CRP. All the top 10 features that showed the highest mean importance among the three best-performing models also had p-values less than 0.001, confirming their significant association with MAFLD prediction. Although it should be noted that in the statistical tests presented in Tables 1 and 2, most features showed p-values below 0.001. This may partly reflect the data preparation process, during which irrelevant variables were removed based on expert opinion. As a result, many of the retained features were already statistically associated with the target variable, which likely reduced the incremental impact of feature selection methods. In addition, the inherent ability of ensemble models to handle redundant or less informative features further limited the benefits of explicit feature selection. Consistently, non-ensemble classifiers such as KNN, which are more sensitive to irrelevant features, showed only modest improvement (approximately 4%) after feature selection.

Before discussing specific biomarkers, it is important to clarify that this study is framed within the MAFLD definition, several earlier studies cited in this section were conducted before the recent redefinition of fatty liver disease and therefore used the term NAFLD. Because all participants in our dataset had type 2 diabetes, a defining metabolic condition under the MAFLD criteria, our cohort fully meets the diagnostic definition of MAFLD. Accordingly, while our analysis refers to MAFLD, comparisons with earlier NAFLD studies are presented using the terminology employed in the original reports to maintain accuracy and historical consistency.

ALT is commonly used in laboratory tests as an indicator of NAFLD, and its significance has been corroborated in various related studies in patients with T2DM [40,41] and other patients [42–45]. However, in other studies predicting MAFLD in patients with T2DM, PLT has not been emphasized significantly. Some studies, such as those by Tomassetti et al., did not find a notable relationship between NAFLD and platelet count [46]. In contrast, most research has found a negative association, which differs from the findings of the current study [47].

For Weight and obesity, more studies have been conducted because of their strong relationship with MAFLD. Published studies have shown that the risk of MAFLD in obese people is 51%, and as previously mentioned, the overall prevalence of MAFLD in the world is estimated to be approximately 25% [48]. Razmpour et al. successfully developed a predictive model for NAFLD with an AUC of 84% using only anthropometric and body composition indices [49].

Many studies have employed statistical tests to examine and establish the effectiveness of various factors in predicting the presence of MAFLD in T2DM patients. Notably, Zheng et al. investigated the correlation of Vit D and found that a low level of Vit D can be an indicator of MAFLD, as was also found in our study [21] which agrees with the outcome of this research.

CRP, as the fourth important feature, is increasingly recognized as a marker of liver inflammation and has been linked to NAFLD, MAFLD, and particularly Non-alcoholic Steatohepatitis (NASH), which is a more advanced stage of these conditions. Additionally, this relationship has been observed in studies focused on T2DM patients with MAFLD [48,50,51].

A key advantage of this study is its use of diverse ensemble and basic ML models, as opposed to the narrower reliance on statistical tests or only logistic regression in prior research [40,41,52,53]. While logistic regression performed well, ensemble methods yielded stronger results. Also, this study focused on the imputation process for making better imputation methods and more accurate and reliable analyses. In this study several feature selection methods were compared, and the results showed that they had limited impact on model performance, as ensemble models inherently manage feature weighting and redundancy and the dataset already contained a set of relevant variables. Nevertheless, applying these methods helped confirm the robustness of the selected predictors and improved interpretability, while slightly reducing the number of required inputs and thus enhancing clinical practicality.

In terms of performance, the best model achieved an AUC of 0.889 surpassing previous studies with AUC values of 0.84 and 0.809 [22,40] But it didn’t surpassed Ye et al.’s study, which achieved an AUC of 90.9% [54]. It is important to note that this study utilized readily available medical and demographic factors collected during routine check-ups of T2DM patients. In contrast, Ye et al. relied on miRNA values, which are typically obtained less frequently and may not be as readily accessible.

This study encountered several limitations, the study was conducted at a single tertiary center and lacks external validation; therefore, generalizability to other institutions and laboratory calibration settings remains to be established. The data were retrospectively collected and cross-sectional, and ultrasound examinations were performed by multiple radiologists without a formal inter-observer agreement assessment, which may introduce label variability. While Little’s test rejected MCAR and operational factors supported a MAR assumption, the missing-data mechanism cannot be proven, and imputation, together with the exclusion of variables with very high missingness, may still add uncertainty. Feature-importance patterns were broadly consistent across the three best-performing models, but relative importances remain model-dependent. Finally, the models were developed on structured clinical and laboratory data only; future work should incorporate multi-center cohorts, leave-hospital-out or external validation, standardized ultrasound grading with inter-rater reliability, and potentially lightweight attention mechanisms or multimodal inputs to enhance robustness and real-world applicability.

Despite these limitations, the primary objective of this study was to enhance the prediction and diagnosis of MAFLD in T2DM patients using readily available routine blood tests and demographic data.

## Conclusion

Overall, this study demonstrates that ML models, particularly ensemble methods, can effectively predict MAFLD in T2DM patients using routine blood tests and demographic data, aiming to provide a practical screening tool for medical staff. This study achieved an AUC of 88.9%. Additionally, a novel approach was applied to evaluate different imputation methods for handling missing data, alongside a comparison of various feature selection techniques. Key predictors such as ALT, PLT, VitD and Weight, which are commonly recorded in standard clinical practice, underscore the feasibility of early screening and intervention, particularly in resource-limited settings. Despite limitations related to missing data and the reliance on ultrasound-based diagnoses, this approach highlights the potential to enhance MAFLD detection and management.

For future studies, it is recommended to consider incorporating lifestyle information of patients into the prediction process. Additionally, obtaining data over specific time intervals may offer an opportunity to discern patterns and enable time series research aimed at predicting the likelihood of MAFLD before its occurrence. Also in our study class imbalance was mild; our comparison of random undersampling and SMOTE showed negligible differences which can be seen in Supplementary S9 and S10 Tables, yet alternative balancing strategies could yield different effects in other cohorts. Furthermore, incorporating lightweight attention mechanisms, such as those proposed in the Attention GhostUNet++ architecture, could help develop compact and efficient deep‐learning pipelines for real‐time MAFLD screening or multimodal feature fusion at the point of care [55].

## Supporting information

S1 Table Full list of 67 original variables and reasons for exclusion or retention. (DOCX)

S2 Table Sensitivity analysis of cohort characteristics under different missingness thresholds (30%, 45%, and 60%). (DOCX)

S3 Table Summary of machine learning models used in this study. (DOCX)

S4 Table Definitions and formulae of evaluation metrics. (DOCX)

S5 Table Hyperparameter grids and best configurations for imputation models. (DOCX)

S6 Table Hyperparameter grids and best configurations for classification models. (DOCX)

S7 Table Feature importance values across top-performing ensemble models. (DOCX)

S8 Table Average performance of imputation models on selected key variables, computed across five independent repetitions with different random seeds. (DOCX)

S9 Table Comparison of model performance under random undersampling. (DOCX)

S10 Table Comparison of model performance using SMOTE. (DOCX)
