# Supplementary material for: Machine learning prediction of metabolic-associated fatty liver disease in type 2 diabetes: Emphasizing data imputation and feature selection
Source: PLoS One. 2026 Feb 24;21(2):e0339580. doi: 10.1371/journal.pone.0339580 (PMC12931757; doi:10.1371/journal.pone.0339580)
Supplement: S1 Table — (DOCX) [file pone.0339580.s001.docx]

**Table S1.Full list of 67 original variables and reasons for exclusion or retention**

| **Variable name** | **Abbreviation / description** | **Reason for exclusion / retention** |
| --- | --- | --- |
| Age | Age (years) | Retained (Final) |
| ALKP | Alkaline phosphatase (U/L) | Retained (Final) |
| ALT | Alanine aminotransferase (U/L) | Retained (Final) |
| APOA | Apolipoprotein A | Removed due to > 40 % missingness |
| APOB | Apolipoprotein B | Removed due to > 40 % missingness |
| AST | Aspartate aminotransferase (U/L) | Retained (Final) |
| BMI | Body Mass Index (kg/m²) | Retained (Final) |
| CABG | Coronary artery bypass graft | Removed by expert (info included in CAD) |
| CAD | Coronary artery disease | Retained (Final) |
| C_peptid | C-peptide (ng/mL) | Removed due to > 40 % missingness |
| CHF | Congestive heart failure | Removed by expert (info included in CAD) |
| CHL | Total cholesterol (mg/dL) | Retained (Final) |
| CRP | C-reactive protein (mg/L) | Retained (Final) |
| Cr | Creatinine (mg/dL) | Retained (Final) |
| CVA | Cerebrovascular accident (stroke) | Retained (Final) |
| DATcad | Year of CAD diagnosis (derived variable) | Removed by expert (unrelevant) |
| DATCABG | Year of CABG procedure (derived variable) | Removed by expert (unrelevant) |
| DATCHF | Year of CHF diagnosis (derived variable) | Removed by expert (unrelevant) |
| DATCVA | Year of CVA diagnosis (derived variable) | Removed by expert (unrelevant) |
| DATdm | Year of diabetes diagnosis (derived variable) | Removed by expert (unrelevant) |
| DATfat | Derived indicator for fatty liver | Removed by expert (unrelevant) |
| DATMI | Year of myocardial infarction (derived) | Removed by expert (info included in CAD) |
| DATMI2 | Secondary indicator for MI (derived) | Removed by expert (unrelevant) |
| DATPCI | Year of PCI procedure (derived) | Removed by expert (unrelevant) |
| DATPCI2 | Secondary indicator for PCI (derived) | Removed by expert (unrelevant) |
| DBP | Diastolic blood pressure (mmHg) | Retained (Final) |
| Dead | Mortality status | Removed due to > 40 % missingness |
| DDM | Duration of diabetes (years) | Retained (Final) |
| Dhtn | Derived code for hypertension | Removed by expert (unrelevant) |
| Drugdm | Diabetes medication (text variable) | Removed (text data) |
| Druglip | Lipid-lowering medication (text variable) | Removed (text data) |
| FBS | Fasting blood sugar (mg/dL) | Retained (Final) |
| FDM | Family history of diabetes mellitus | Removed by expert (unrelevant) |
| FHcad | Family history of CAD | Removed by expert (unrelevant) |
| FHhlp | Family history of hyperlipidemia | Removed by expert (unrelevant) |
| FHhtn | Family history of hypertension | Removed by expert (unrelevant) |
| GGT | Gamma-glutamyl transferase (U/L) | Removed due to > 40 % missingness |
| Grade of fatty liver | Fatty liver grading (stage) | Target variable (not used in model) |
| HB | Hemoglobin (g/dL) | Removed due to > 40 % missingness |
| HDL | High-density lipoprotein (mg/dL) | Retained (Final) |
| Height | Height (cm) | Retained (Final) |
| High | Hypertension (Yes/No) | Retained (Final) |
| Hip | Hip circumference (cm) | Retained (Final) |
| HOMA | Homeostatic Model Assessment index | Retained (Final) |
| HPP | 2-hour postprandial plasma glucose (mg/dL) | Retained (Final) |
| HTN | Hypertension diagnosis (Yes/No) | Retained (Final) |
| Insulin | Fasting insulin (µIU/mL) | Retained (Final) |
| LDL | Low-density lipoprotein (mg/dL) | Retained (Final) |
| LPA | Lipoprotein A | Removed due to > 40 % missingness |
| Microalb | Urinary microalbumin (mg/L) | Removed by expert (unrelevant) |
| Microcat | Microalbumin category | Removed by expert (unrelevant) |
| MI | Myocardial infarction (history) | Removed by expert (info included in CAD) |
| PCI | Percutaneous coronary intervention (history) | Removed by expert (info included in CAD) |
| PCI3 | Third indicator for PCI (derived) | Removed by expert (info included in CAD) |
| PLT | Platelet count (cells/µL) | Retained (Final) |
| Retino | Diabetic retinopathy (Yes/No) | Retained (Final) |
| SBP | Systolic blood pressure (mmHg) | Removed by expert (wrong correlation with DBP) |
| Sex | Biological sex (Male/Female) | Retained (Final) |
| Smoking | Smoking status (Yes/No) | Retained (Final) |
| TG | Triglycerides (mg/dL) | Retained (Final) |
| UA | Uric acid (mg/dL) | Retained (Final) |
| UREA | Blood urea nitrogen (mg/dL) | Removed by expert (unrelevant) |
| VitD | Vitamin D (ng/mL) | Retained (Final) |
| Waist | Waist circumference (cm) | Retained (Final) |
| Weight | Weight (kg) | Retained (Final) |
| Fatty liver | Fatty liver presence (MAFLD target) | Target variable (not used as predictor) |
