# Supplementary material for: Machine learning prediction of metabolic-associated fatty liver disease in type 2 diabetes: Emphasizing data imputation and feature selection
Source: PLoS One. 2026 Feb 24;21(2):e0339580. doi: 10.1371/journal.pone.0339580 (PMC12931757; doi:10.1371/journal.pone.0339580)
Supplement: S2 Table — (DOCX) [file pone.0339580.s002.docx]

**Table S2. Sensitivity analysis of cohort characteristics under different missingness thresholds (30%, 45%, and 60%)**

| **Feature** | **Scenario Compared to Base** | **Standardized Mean Difference** | **Kolmogorov–Smirnov p-value** | **base_missing** | **alt_missing** |
| --- | --- | --- | --- | --- | --- |
| DDM | S1_30_30 | 0.002287173 | 1 | 0.005309735 | 0.004839531 |
| Retino | S1_30_30 | 0.000453451 | 1 | 0.07863464 | 0.077432501 |
| HTN | S1_30_30 | -0.00380627 | 1 | 0.003034134 | 0.003056546 |
| FHHTN | S1_30_30 | 0.000777961 | 1 | 0.100379267 | 0.097300051 |
| FHCAD | S1_30_30 | -0.001250963 | 1 | 0.100379267 | 0.096790627 |
| Sex | S1_30_30 | 0.002242044 | 1 | 0 | 0 |
| Age | S1_30_30 | 0.004178091 | 1 | 0 | 0 |
| High | S1_30_30 | 0.000117284 | 1 | 0.000252845 | 0.000254712 |
| Weight | S1_30_30 | -0.004848414 | 1 | 0.000252845 | 0.000254712 |
| Waist | S1_30_30 | -0.002835264 | 1 | 0.001517067 | 0.000764137 |
| Hip | S1_30_30 | -0.005426662 | 1 | 0.09608091 | 0.092715232 |
| SBP | S1_30_30 | -0.001626041 | 1 | 0 | 0 |
| DBP | S1_30_30 | -0.002806892 | 1 | 0 | 0 |
| VitD | S1_30_30 | 1.16365E-05 | 1 | 0.15954488 | 0.153591442 |
| FBS | S1_30_30 | -0.003993325 | 1 | 0.000252845 | 0 |
| HPP | S1_30_30 | -0.002565371 | 1 | 0.009860936 | 0.007641365 |
| HBA1C | S1_30_30 | -0.000186962 | 1 | 0.006573957 | 0.004584819 |
| Insulin | S1_30_30 | -0.002402885 | 1 | 0.096333755 | 0.092969944 |
| CHL | S1_30_30 | -0.003396331 | 1 | 0.001517067 | 0.000254712 |
| HDL | S1_30_30 | 0.001339356 | 1 | 0.001769912 | 0.000254712 |
| LDL | S1_30_30 | -0.001834506 | 1 | 0.00278129 | 0.000509424 |
| TG | S1_30_30 | -0.007477572 | 1 | 0.002275601 | 0.001018849 |
| Cr | S1_30_30 | -0.000450663 | 1 | 0.001517067 | 0.000509424 |
| UA | S1_30_30 | 0.00038462 | 1 | 0.063716814 | 0.057564952 |
| AST | S1_30_30 | -0.000159708 | 1 | 0.026548673 | 0.021141111 |
| ALT | S1_30_30 | -4.25414E-05 | 1 | 0.023261694 | 0.018084564 |
| ALP | S1_30_30 | 6.55897E-05 | 1 | 0.21517067 | 0.209882832 |
| CAD | S1_30_30 | -0.002642716 | 1 | 0.058154235 | 0.057819664 |
| CABG | S1_30_30 | -0.005757652 | 1 | 0.185082174 | 0.181609781 |
| MI | S1_30_30 | -0.002864972 | 1 | 0.195701643 | 0.191543556 |
| PCI | S1_30_30 | -0.00058087 | 1 | 0.194437421 | 0.190524707 |
| CVA | S1_30_30 | -0.001918986 | 1 | 0.21289507 | 0.208609272 |
| Smoking | S1_30_30 | 0.001133506 | 1 | 0.225031606 | 0.223382578 |
| DDM | S3_60_60 | 9.83139E-05 | 1 | 0.005309735 | 0.005300353 |
| PLT | S3_60_60 | -0.000774301 | 1 | 0.402528445 | 0.403079253 |
| Retino | S3_60_60 | 0.000169312 | 1 | 0.07863464 | 0.078495709 |
| HTN | S3_60_60 | 0.000607798 | 1 | 0.003034134 | 0.003028773 |
| FHHTN | S3_60_60 | -0.000220599 | 1 | 0.100379267 | 0.101716305 |
| FHCAD | S3_60_60 | -0.000164578 | 1 | 0.100379267 | 0.101716305 |
| Sex | S3_60_60 | 0.000388843 | 1 | 0 | 0 |
| Age | S3_60_60 | 0.00078731 | 1 | 0 | 0 |
| High | S3_60_60 | -2.81169E-05 | 1 | 0.000252845 | 0.000252398 |
| Weight | S3_60_60 | -0.000315313 | 1 | 0.000252845 | 0.000252398 |
| Waist | S3_60_60 | -0.000143347 | 1 | 0.001517067 | 0.001514387 |
| Hip | S3_60_60 | -5.44743E-05 | 1 | 0.09608091 | 0.097425543 |
| SBP | S3_60_60 | 0.000340043 | 1 | 0 | 0 |
| DBP | S3_60_60 | -0.00135113 | 1 | 0 | 0 |
| CRP | S3_60_60 | 0 | 1 | 0.360809102 | 0.361938415 |
| VitD | S3_60_60 | 8.88312E-05 | 1 | 0.15954488 | 0.160777385 |
| FBS | S3_60_60 | 1.99462E-05 | 1 | 0.000252845 | 0.001514387 |
| HPP | S3_60_60 | 0 | 1 | 0.009860936 | 0.011610298 |
| HBA1C | S3_60_60 | 0 | 1 | 0.006573957 | 0.008329127 |
| Insulin | S3_60_60 | -0.000291832 | 1 | 0.096333755 | 0.09767794 |
| CHL | S3_60_60 | 9.78843E-05 | 1 | 0.001517067 | 0.003028773 |
| HDL | S3_60_60 | 0 | 1 | 0.001769912 | 0.003533569 |
| LDL | S3_60_60 | 0 | 1 | 0.00278129 | 0.00454316 |
| TG | S3_60_60 | -0.000203497 | 1 | 0.002275601 | 0.003785967 |
| Cr | S3_60_60 | -1.78389E-06 | 1 | 0.001517067 | 0.003028773 |
| UA | S3_60_60 | 0 | 1 | 0.063716814 | 0.065371025 |
| AST | S3_60_60 | 0 | 1 | 0.026548673 | 0.028268551 |
| ALT | S3_60_60 | 0 | 1 | 0.023261694 | 0.02498738 |
| ALP | S3_60_60 | 0 | 1 | 0.21517067 | 0.216557294 |
| CAD | S3_60_60 | 0.002454207 | 1 | 0.058154235 | 0.058051489 |
| CHF | S3_60_60 | 0 | 1 | 0.308217446 | 0.309439677 |
| CABG | S3_60_60 | 0.00395036 | 1 | 0.185082174 | 0.18525997 |
| MI | S3_60_60 | -0.000312142 | 1 | 0.195701643 | 0.195860676 |
| PCI | S3_60_60 | 0.000150149 | 1 | 0.194437421 | 0.194598688 |
| CVA | S3_60_60 | -0.000239693 | 1 | 0.21289507 | 0.213023725 |
| Smoking | S3_60_60 | 0.001151548 | 1 | 0.225031606 | 0.224886421 |

Base Scenario: Column threshold = 45%, Row threshold = 50%, S1_30_30: Column threshold = 30%, Row threshold = 30%, S3_60_60: Column threshold = 60%, Row threshold = 60%
