# Supplementary material for: Machine learning prediction of metabolic-associated fatty liver disease in type 2 diabetes: Emphasizing data imputation and feature selection
Source: PLoS One. 2026 Feb 24;21(2):e0339580. doi: 10.1371/journal.pone.0339580 (PMC12931757; doi:10.1371/journal.pone.0339580)
Supplement: S3 Table — (DOCX) [file pone.0339580.s003.docx]

**Table S3.Summary of machine learning models used in this study**

| **Model** | **Description** |
| --- | --- |
| Ridge Regression | Ridge regression is a type of linear regression that incorporates L2 regularization to mitigate the effect of multicollinearity by penalizing large coefficient values. This penalty shrinks the regression coefficients toward zero, preventing them from becoming excessively large and reducing overfitting. |
| Logistic Regression | Logistic Regression is a linear classification model that estimates the probability of class membership by modeling the log-odds as a linear function of the input features. The model ensures that predicted probabilities remain within the range [0,1] and sum to one across all classes. Due to its simplicity, interpretability, and strong theoretical foundation, logistic regression serves as a standard baseline for classification problems ^36^. |
| k-Nearest Neighbors | KNN algorithm is a non-parametric classification method that assigns labels to new observations based on the majority class among the k closest training samples. The similarity between observations is typically measured using a distance metric. Because KNN does not make any assumptions about data distribution, it is highly flexible and applicable to various datasets, though its computational costs can be significant with large datasets ^37^. |
| Support Vector Classifier | Support Vector Classifier is an implementation of Support Vector Machines (SVM) designed for classification. It works by identifying the optimal hyperplane that maximizes the margin between different classes in the feature space. When classes aren't linearly separable, SVC utilizes kernel functions to project data into a higher-dimensional space where a separating hyperplane can be found. This allows SVC to effectively manage high-dimensional data and complex class boundaries ^38^. |
| Decision Tree | Decision Tree is hierarchical models that recursively split the feature space into distinct regions based on feature values. The model selects the best splitting feature at each node to minimize impurity measures such as Gini impurity or entropy. Decision trees are highly interpretable and can capture non-linear relationships, but they are prone to overfitting if not properly regularized ^37^. |
| Random Forest | Random Forest is an ensemble learning method that constructs multiple decision trees using bootstrap sampling and random feature selection. By aggregating predictions from multiple trees (through majority vote in classification tasks or by computing the mean of predictions in regression tasks), it reduces variance and mitigates overfitting. RF is widely used due to its robustness, ability to handle high-dimensional data, and strong generalization performance ^39, 40^. |
| Extremely Randomized Trees | Extremely Randomized Trees is an ensemble method like Random Forest but introduces additional randomness in the tree construction process. Unlike Random Forest, which selects the best feature split based on information gain, Extra Trees selects split thresholds randomly. This approach results in lower variance and computational efficiency while maintaining high predictive performance ^40^. |
| Adaptive Boosting | AdaBoost is a boosting algorithm that sequentially trains a series of weak classifiers, typically decision trees, and assigns greater weight to misclassified samples in each iteration. By focusing on difficult cases, AdaBoost builds a strong classifier with improved overall accuracy. It has been widely applied in domains such as object recognition and fraud detection ^41^. |
| Gradient Boosting | GB is another boosting technique that constructs models sequentially, optimizing a given loss function using gradient descent. Unlike AdaBoost, which adjusts sample weights, Gradient Boosting fits new decision trees to residual errors of previous models. This iterative learning process allows the model to minimize prediction errors effectively. However, GB can be sensitive to noise and prone to overfitting without proper tuning. |
| Extreme Gradient Boosting | XGBoost is an optimized gradient boosting framework that improves performance with parallel processing, regularization methods, and sophisticated tree-pruning strategies. It is particularly known for its scalability and superior predictive performance, making it a dominant method in machine learning competitions. XGBoost effectively handles large datasets and missing values while reducing the risk of overfitting ^40^. |
| LightGBM | LightGBM is an efficient gradient boosting algorithm that employs a leaf-wise tree growth strategy instead of a level-wise approach. This method results in deeper trees, improved accuracy, and faster training times while maintaining low memory usage. LightGBM is highly effective for high-dimensional data and large-scale machine learning tasks, which has made it widely preferred in both research and industry applications. ^40^. |
