# Supplementary material for: Machine learning prediction of metabolic-associated fatty liver disease in type 2 diabetes: Emphasizing data imputation and feature selection
Source: PLoS One. 2026 Feb 24;21(2):e0339580. doi: 10.1371/journal.pone.0339580 (PMC12931757; doi:10.1371/journal.pone.0339580)
Supplement: S4 Table — (DOCX) [file pone.0339580.s004.docx]

**Table S4. Definitions and formulae of evaluation metrics**

| **Model** | **Description** | **Formula** |
| --- | --- | --- |
| Accuracy | Accuracy is the ratio of the number of correctly predicted records to the total number of records. | $\frac{TN+TP}{TN+FP+FN+TP}$ |
| Precision | Precision quantifies the proportion of true positive predictions among all records predicted as positive, indicating its ability to reduce false positives. | $\frac{TP}{TP+FP}$ |
| Recall | Recall is the ratio of correctly identified positives to all real positives in the data. | $\frac{TN}{TN+FP}$ |
| F1 Score | The F1 score is the harmonic mean of precision and recall, offering a balanced metric that considers both the model’s accuracy in making positive predictions and its ability to identify all relevant instances. | $\frac{2}{\frac{1}{Precision}+ \frac{1}{Recall}}$ |
| Area Under the ROC Curve (AUC) | AUC evaluates a model's ability to distinguish between classes across various threshold settings. A higher AUC value indicates the model's better ability to differentiate between the positive and negative classes |  |
| R squared | R^2^ measures the proportion of variance in the dependent variable that is predictable from the independent variables. It provides an indication of the model’s goodness of fit. | $1- \frac{Sum Squared Regression Error}{Sum Squared Total Error}$ |
