# Supplementary material for: Machine learning prediction of metabolic-associated fatty liver disease in type 2 diabetes: Emphasizing data imputation and feature selection
Source: PLoS One. 2026 Feb 24;21(2):e0339580. doi: 10.1371/journal.pone.0339580 (PMC12931757; doi:10.1371/journal.pone.0339580)
Supplement: S5 Table — (DOCX) [file pone.0339580.s005.docx]

**Table S5. Hyperparameter grids and best configurations for imputation models**

| **Imputation Model** | **Key Hyperparameters** |
| --- | --- |
| AdaBoost | 'n_estimators'= 200, 'learning_rate'= 0.1 |
| DecisionTree | 'max_depth'= 3, 'min_samples_split'= 5 |
| ExtraTrees | 'n_estimators'= 100, 'max_depth'= 5 |
| KNN | 'n_neighbors'= 15 |
| MissForest | 'maxiter'= 5, 'ntree'= 50 |
| RandomForest | 'n_estimators'= 100, 'max_depth'= 5 |
| Ridge | 'fit_intercept'= True, 'copy_X'= True, 'alpha'= 10.0 |
| SVR | 'C'= 10 |
| XGBoost | 'max_depth'= 3, 'learning_rate'= 0.05 |
