# Supplementary material for: Machine learning prediction of metabolic-associated fatty liver disease in type 2 diabetes: Emphasizing data imputation and feature selection
Source: PLoS One. 2026 Feb 24;21(2):e0339580. doi: 10.1371/journal.pone.0339580 (PMC12931757; doi:10.1371/journal.pone.0339580)
Supplement: S6 Table — (DOCX) [file pone.0339580.s006.docx]

**Table S6. Hyperparameter grids and best configurations for classification models**

| **Model** | **Key Hyperparameters** |
| --- | --- |
| Logistic Regression | C = 1, solver = ‘liblinear’, max_iter = 1000, random_state = 0 |
| KNN | n_neighbors = 7, weights = ‘distance’ |
| SVM | C = 1, kernel = ‘rbf’ (default), probability = True, random_state = 0 |
| Decision Tree | max_depth = 5, min_samples_split = 5, random_state = 0 |
| Extra Trees | n_estimators = 300, max_depth = None, min_samples_split = 2, n_jobs = –1, random_state = 0 |
| Gradient Boosting | n_estimators = 300, learning_rate = 0.1, max_depth = 5, random_state = 0 |
| XGBoost | n_estimators = 300, learning_rate = 0.1, max_depth = 5, subsample = 1.0, colsample_bytree = 1.0, eval_metric = ‘logloss’, tree_method = ‘hist’, n_jobs = –1, random_state = 0 |
| LightGBM | n_estimators = 100, learning_rate = 0.1, max_depth = 7, num_leaves = 31, force_col_wise = True, n_jobs = –1, random_state = 0 |
