# Supplementary material for: Machine learning prediction of metabolic-associated fatty liver disease in type 2 diabetes: Emphasizing data imputation and feature selection
Source: PLoS One. 2026 Feb 24;21(2):e0339580. doi: 10.1371/journal.pone.0339580 (PMC12931757; doi:10.1371/journal.pone.0339580)
Supplement: S7 Table — (DOCX) [file pone.0339580.s007.docx]

**Table S7. Feature importance values across top-performing ensemble models**

| **Features** | **LightGBM** | **Gradient Boosting** | **XGBoost** |
| --- | --- | --- | --- |
| ALT | 0.077435 | 0.336715 | 0.1876 |
| PLT | 0.138052 | 0.114301 | 0.049 |
| VitD | 0.092151 | 0.053771 | 0.0317 |
| Weight | 0.060967 | 0.048345 | 0.0517 |
| CRP | 0.073231 | 0.041673 | 0.0337 |
| ALKP | 0.070778 | 0.032348 | 0.02486 |
| CHL | 0.071479 | 0.026092 | 0.0258 |
| HOMA | 0.066573 | 0.024964 | 0.02132 |
| DDM | 0.053609 | 0.031388 | 0.02712 |
| TG | 0.061668 | 0.027736 | 0.021436 |
| Waist | 0.039944 | 0.015138 | 0.05173 |
| Hip | 0.046601 | 0.024168 | 0.03077 |
| High | 0.042046 | 0.025629 | 0.0298 |
| FBS | 0.058865 | 0 | 0.0252 |
| DBP | 0.02768 | 0.018025 | 0.0309 |
| BMI | 0 | 0.036108 | 0.0343 |
| Sex | 0.010862 | 0.009751 | 0.049 |
| UA | 0 | 0.030437 | 0.0284 |
| Insulin | 0 | 0.028446 | 0.02908 |
| Age | 0 | 0.018947 | 0.02488 |
| LDL | 0 | 0.018417 | 0.02339 |
| AST | 0 | 0.019056 | 0.02152 |
| HDL | 0 | 0.018545 | 0.01783 |
| Retino | 0.002453 | 0 | 0.02833 |
| CAD | 0.005606 | 0 | 0.0247 |
| Cr | 0 | 0 | 0.02354 |
| HPP | 0 | 0 | 0.02075 |
| HBA1C | 0 | 0 | 0.02023 |
| HTN | 0 | 0 | 0.0172 |
| CVA | 0 | 0 | 0.01551 |
| Smoking | 0 | 0 | 0.0096 |
