# Supplementary material for: Machine learning prediction of metabolic-associated fatty liver disease in type 2 diabetes: Emphasizing data imputation and feature selection
Source: PLoS One. 2026 Feb 24;21(2):e0339580. doi: 10.1371/journal.pone.0339580 (PMC12931757; doi:10.1371/journal.pone.0339580)
Supplement: S8 Table — (DOCX) [file pone.0339580.s008.docx]

**Table S8. Average performance of imputation models on selected key variables, computed across five independent repetitions with different random seeds.**

| **Imputation Model** | **R^2^ PLT** | **R^2^ HOMA** | **R^2^ VitD** | **R^2^ ALT** | **Accuracy Retino** | **R^2^ CRP** |
| --- | --- | --- | --- | --- | --- | --- |
| AdaBoost | 0.023 | 0.621 | 0.005 | 0.490 | 0.924 | 0.042 |
| DecisionTree | -0.170 | 0.428 | -0.437 | 0.402 | 0.892 | -0.813 |
| ExtraTrees | 0.012 | 0.663 | 0.011 | 0.479 | 0.924 | -0.155 |
| KNN | 0.019 | 0.139 | -0.034 | 0.069 | 0.920 | -0.224 |
| MissForest | 0.009 | 0.328 | -0.008 | 0.451 | 0.920 | -0.155 |
| RandomForest | 0.006 | 0.739 | -0.066 | 0.465 | 0.924 | -0.453 |
| Ridge | -0.119 | 0.759 | -0.025 | 0.488 | 0.920 | -0.384 |
| SVR | 0.004 | 0.550 | -0.020 | 0.173 | 0.920 | -0.066 |
| XGBoost | 0.014 | 0.639 | 0.014 | 0.454 | 0.916 | -0.140 |
