# Supplementary material for: Machine learning prediction of metabolic-associated fatty liver disease in type 2 diabetes: Emphasizing data imputation and feature selection
Source: PLoS One. 2026 Feb 24;21(2):e0339580. doi: 10.1371/journal.pone.0339580 (PMC12931757; doi:10.1371/journal.pone.0339580)
Supplement: S9 Table — (DOCX) [file pone.0339580.s009.docx]

**Table S9. Comparison of model performance under random undersampling**

| **Row Labels** | **Accuracy** | **Recall** | **Precision** | **F1** | **AUC** |
| --- | --- | --- | --- | --- | --- |
| Logistic Regression | 76.5% | 72.9% | 78.6% | 75.6% | 83.6% |
| KNN | 72.4% | 65.7% | 75.9% | 70.4% | 79.8% |
| SVM | 76.8% | 76.0% | 77.3% | 76.6% | 84.3% |
| Decision Tree | 74.7% | 71.2% | 76.8% | 73.8% | 81.3% |
| Extra Tree | 79.0% | 76.8% | 80.3% | 78.5% | 87.3% |
| Gradient Boosting | 80.3% | 79.4% | 80.9% | 80.1% | 88.7% |
| XGBoost | 80.6% | 79.6% | 81.2% | 80.4% | 88.9% |
| LightGBM | 80.3% | 79.0% | 81.1% | 80.0% | 88.8% |
