# Supplementary material for: Machine learning prediction of metabolic-associated fatty liver disease in type 2 diabetes: Emphasizing data imputation and feature selection
Source: PLoS One. 2026 Feb 24;21(2):e0339580. doi: 10.1371/journal.pone.0339580 (PMC12931757; doi:10.1371/journal.pone.0339580)
Supplement: S10 Table — (DOCX) [file pone.0339580.s010.docx]

**Table S10. Comparison of model performance SMOTE**

| **Row Labels** | **Accuracy** | **Recall** | **Precision** | **F1** | **AUC** |
| --- | --- | --- | --- | --- | --- |
| Logistic Regression | 76.60% | 73.10% | 78.00% | 75.40% | 83.70% |
| KNN | 72.60% | 66.20% | 75.10% | 70.30% | 79.80% |
| SVM | 76.70% | 75.50% | 76.70% | 76.10% | 84.30% |
| Decision Tree | 74.90% | 73.20% | 75.10% | 74.00% | 81.60% |
| Extra Tree | 79.00% | 76.50% | 79.90% | 78.10% | 87.30% |
| Gradient Boosting | 80.60% | 79.10% | 81.10% | 80.00% | 89.10% |
| XGBoost | 80.80% | 79.20% | 81.30% | 80.20% | 89.30% |
| LightGBM | 80.90% | 79.40% | 81.30% | 80.30% | 89.00% |
